# SEM Evaluation of Tooth Surface after a Composite Filling Removal Using Er:YAG Laser, Drills with and without Curettes, and Optional EDTA or NaOCl Conditioning

**DOI:** 10.3390/ma14164469

**Published:** 2021-08-10

**Authors:** Jan Kiryk, Jacek Matys, Kinga Grzech-Leśniak, Marzena Dominiak, Małgorzata Małecka, Piotr Kuropka, Rafał J. Wiglusz, Maciej Dobrzyński

**Affiliations:** 1 Dental Surgery Department, Medical University of Wroclaw, 50-425 Wroclaw, Poland; jan.kiryk@umed.wroc.pl (J.K.); marzena.dominiak@umed.wroc.pl (M.D.); 2 Laser Laboratory at Dental Surgery Department, Wroclaw Medical University, Krakowska 26, 50-425 Wroclaw, Poland; 3 Department of Periodontics School of Dentistry, Virginia Commonwealth University, VCU, Richmond, VA 23298, USA; 4 Institute of Low Temperature and Structure Research, Polish Academy of Sciences, Okolna 2, 50-422 Wroclaw, Poland; m.malecka@intibs.pl; 5 Department of Histology and Embriology, Wroclaw University of Environmental and Life Sciences, Norwida 31, 50-375 Wroclaw, Poland; piotr.kuropka@upwr.edu.pl; 6 Department of Pediatric Dentistry and Preclinical Dentistry, Wroclaw Medical University, Krakowska 26, 50-425 Wroclaw, Poland; maciej.dobrzynski@umed.wroc.pl

**Keywords:** CEJ, collagen fibers, dentinal tubules, gingival recession, smear layer

## Abstract

(1) Background: This study aimed to evaluate the microporosity of the tooth surface structure adjacent to the cemento-enamel junction (CEJ) after the removal of composite fillings with a drill in comparison with removal by an Er:YAG laser and after cleaning with a periodontal curette, chemical EDTA and NaOCl (sodium hypochlorite) conditioning. (2) Methods: The research material consisted of 30 extracted premolars with cervical composite fillings. The teeth were divided into six groups according to the method of tooth preparation: group G1 (*n* = 5)—a diamond drill; group G2 (*n* = 5)—a diamond drill + curette; group G3 (*n* = 5)—a diamond drill + 24% EDTA (PrefGel, Straumann, Switzerland); group G4 (*n* = 5)—an Er:YAG laser (LightWalker, Fotona, Ljubljana, Slovenia) set with the following parameters: power: 1.65 W (composite removal, CR), 1.2 (tooth conditioning, TC), energy: 110 mJ (CR), 80 mJ (TC), frequency: 15 Hz, pulse duration: 50 μs, tip diameter: 1 mm, air/fluid cooling: 4, distance 1.5 mm, energy density: 14.01 J/cm^2^ (CR), 10.19 J/cm^2^ (TC); group G5 (*n* = 5)—an Er:YAG laser + 2% sodium hypochlorite (NaOCl); group G6 (*n* = 5)—an Er:YAG laser + 5.25% NaOCl. In each tooth, three cavities were made and subjected to analysis. The dentin surface was evaluated using a scanning electron microscope (SEM). (3) Results: Groups G1 and G2 exhibited mechanical damage to the tooth surface structure caused by the rotary motion of a diamond drill. The SEM image showed a smear layer that could only be removed chemically using 24% EDTA gel (group G3). The tooth surfaces prepared with the Er:YAG laser (groups G4–G6) revealed a homogeneous structure without damage along with open dentinal tubules (without smear layer) and visible denaturation of collagen fibers. The sodium hypochlorite (NaOCl) conditioning did not increase the visibility of dentinal tubules. (4) Conclusions: Dentin surfaces have open dentinal tubules after removal of the composite filling using the Er:YAG laser and therefore do not require additional NaOCl conditioning.

## 1. Introduction

Dental filling removal in the cervical region of the tooth is a procedure that is performed before gingival recession coverage [1]. The presence of the filling in the cemento-enamel junction (CEJ) or on the dental root surface impairs the connection of fibroblasts with dental tissues and, thus, prevents successful gingival recession coverage [2]. Gingival recession can be found in people with very good oral hygiene where it mainly affects labial surface [3,4,5]. Gingival recession can also occur among individuals who exhibit negligence in oral hygiene; in such cases, it affects all tooth surfaces [5]. Restoration of hard-tissue defects in the labial and buccal regions of the teeth without gum regenerative procedures may cause other problems, such as dentin hypersensitivity, root caries and patient discomfort for aesthetic reasons [6,7].

To perform the procedure of gingival recession coverage, the root surface needs to be free of caries and cannot be covered with any dental material or smear layer, as the connective tissue attachment can only be formed properly in contact with the dental tissue [8]. Initially, the tooth should be cleaned of calculus and plaque. The use of hand instruments or ultrasonic instruments forms a smear layer that adheres tightly to the root surface and cannot be removed by conventional water rinsing [9]. Exposed root surfaces in patients with gingival recession may also cause endotoxin contamination [10]. Therefore, many clinicians use root surface bio-modification agents, such as ethylenediaminetetraacetic acid (EDTA), citric acid, sodium hypochlorite (NaOCl) or Er:YAG laser conditioning to remove the smear layer and open the dentinal tubules [11].

When considering all known laser wavelengths, Er:YAG laser radiation at 2940 nm exhibits the highest absorption of water in all layers of hard dental tissues [12,13,14,15,16]. The core (medium) of Er:YAG lasers, which are widely used in conservative dentistry, is an erbium crystal with an admixture of yttrium, aluminum and garnet. The wavelength of 2940 nm is characterized by shallow tissue penetration in the range of several to several dozen micrometers. In addition, the higher percentage of water in caries (compared to a healthy dentin and enamel) influences the selectivity of laser work, leading to faster ablation of sick tissues and assuring safety in relation to the pulp [1]. The prepared cavity has a surface structure resembling a surface conditioned with a 37% orthophosphoric acid but with more significant irregularity [17]. The prepared surface after laser application is decontaminated (microbiologically clean) and it does not display deep mechanical damage, especially when using appropriate laser operating parameters and water spray cooling [18,19]. Importantly, laser preparation is also safe for the dental pulp, as no thermal damage is observed [20,21,22]. It should also be noted that the procedure is well accepted by patients because of less pain and reduced need for topical anesthesia before the procedure [23].

This study aimed to evaluate, by means of a scanning electron microscope (SEM), the microporosity of the tooth surface structure after removal of a composite filling from the cervical region of the tooth (CEJ) when using an Er:YAG laser with additional conditioning of the dentin surface with sodium hypochlorite (NaOCl) compared to that when using a traditional filling removal method with a diamond dental drill and periodontal curette or chemical EDTA conditioning in order to determine their usefulness in procedures for gingival recession coverage.

## 2. Materials and Methods

The research material consisted of 30 premolar teeth with cervical fillings. It was determined from the medical history that the teeth had been restored with composite fillings between 6 months and 7 years prior (with an average duration of 3 years before tooth extraction). The teeth had later been removed at the Dental Surgery Department of Wroclaw Medical University due to orthodontic and periodontal indications. After extraction, the teeth were stored in 1% thymol solution. The storage period for the teeth before the SEM analysis did not exceed 2 days. Three cavities were prepared in each tooth using different methods as described in the below-mentioned paragraph. Sample size was calculated to be 15 (3 cavities multiplied by 5 teeth) in each group using G×Power (Kiel University, Kiel, Germany) software assuming 80% power of study, 95% confidence interval, a level of significance of 0.05 and *d* = 0.94. The study was conducted in line with the approval No. KB 132/2019 issued by the Bioethics Committee appointed by the Rector of Wroclaw Medical University.

### 2.1. Mechanical and Mechanochemical Preparation of Dentin

In Group G1 (Drill, *n* = 5), the composite material was removed by means of a conventional method using a ball-nose diamond drill #014 (Meisinger, Neuss, Germany) with blue coating on a water-cooled turbine tip (NSK, Tokyo, Japan).

In Group G2 (Drill + Curette, *n* = 5), the composite material was removed using a ball-nose diamond drill #014 (Meisinger, Neuss, Germany) with blue coating on a water-cooled turbine tip (NSK, Tokyo, Japan), and the cavity was cleaned with a Gracey ½ curette (Henry Schein, NY, USA).

In Group G3 (Drill + EDTA, *n* = 5), the composite material was removed using a ball-nose diamond drill #014 (Meisinger, Neuss, Germany) with blue coating on a water-cooled turbine tip (NSK, Tokyo, Japan), and the cavity surface was conditioned with a 24% EDTA solution (PrefGel, Straumann, Basel, Switzerland) in accordance with the manufacturer’s recommended protocol (application to the cleaned surface for 2 min and rinsing with distilled water).

In Group G4 (Er:YAG laser, *n* = 5), the composite material was removed using an Er:YAG laser (LightWalker, Fotona, Ljubljana, Slovenia) with the following set parameters: power: 1.65 W (composite removal, CR), 1.2 W (tooth conditioning, TC); energy: 110 mJ (CR), 80 mJ (TC); frequency: 15 Hz; pulse duration: 50 μs; tip diameter: 1 mm; air/fluid cooling: 4; distance: 1.5 mm; energy density: 14.01 J/cm^2^ (CR), 10.19 J/cm^2^ (TC).

In Group G5 (Er:YAG laser + 2%NaOCl, *n* = 5), the composite material was removed using an Er:YAG laser with identical parameters to those of Group G4, while the cavity surface was conditioned with 2% NaOCl solution (CERKAMED, Stalowa Wola, Poland) by rubbing it with the aforementioned solution for 15 s and rinsing with distilled water for 10 s.

In Group G6 (Er:YAG laser + 5.25%NaOCl, *n* = 5), the composite material was removed using an Er:YAG laser with identical parameters to those of Groups G4 and G5, while the cavity surface was conditioned with 5.25% NaOCl solution (CERKAMED, Stalowa Wola, Poland) by rubbing it with the aforementioned solution for 15 s and rinsing with distilled water for 10 s.

### 2.2. Scanning Electron Microscopy

The teeth in each group were fixed in 2.5% glutaraldehyde using a 7.4 phosphate buffer. Subsequently, the samples were rinsed with a phosphate buffer and then dehydrated in an acetone solution series (from 50% to 100%). The teeth were dried and mounted on stubs. The collected material was analyzed using FE-SEM microscope FEI NovaNanoSEM 230 (FEI Company, Hillsboro, OR, USA). SEM settings during the analysis of the tooth surface were as follows: HiVac 2 × 10^−4^ Pa, WD 6.1 to 8.4 mm, 5.00 kV, spot 4.5, magnification 100× or 2000×, 5.00 keV.

### 2.3. Semi-Quantitative Evaluation of Test Samples

The quantitative evaluation was performed according to Attin [24] with our own modifications. The importance of each factor was adapted to the requirements of subsequent procedures. The subjective evaluation made by a histologist (P.K.) took into account the quality of the tooth preparation (0–2 pts) with the most desired regular cavity shape, the exposure of dentinal tubules (0–1 pt), the presence of smear layer (0–1 pt) and the degree of similarity of test samples in a group (0–2 pts). The semi-quantitative evaluation score scale ranged between 0 and 6. Scores above 3 were rated as desirable [25]. The aforementioned method of analysis is one of numerous methods described in the literature [24,25].

### 2.4. Statistical Analysis

The intragroup comparison of the samples evaluated by the semi-quantitative method was analyzed with the Kruskal–Wallis ANOVA test. Statistica software (StatSoft, Tulsa, OK, USA) was used for statistical analysis. Values below *p* = 0.05 were considered to be statistically significant.

## 3. Results

### 3.1. Effects of the Drill and Curette on Dentin Structure

**Figure 1:**
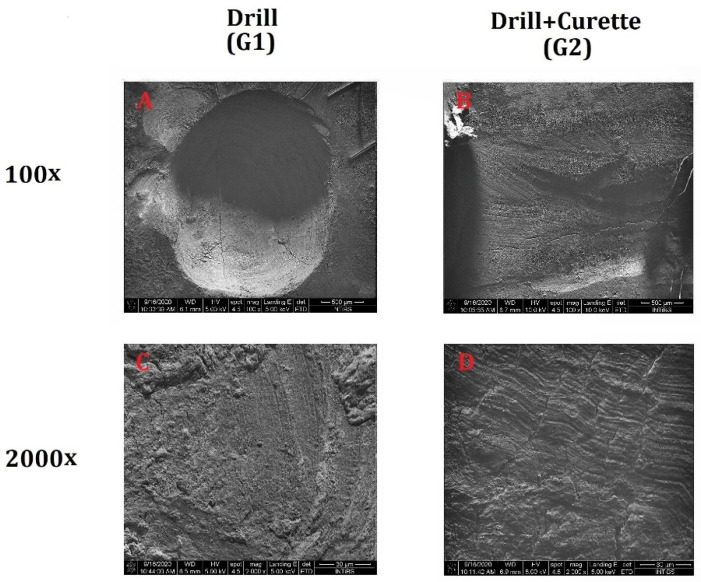
Dentin surface after removal of the composite filling using a diamond drill (**A**,**C**) and after additional cleaning of the dentin using a dental curette (**B**,**D**).

After the composite material was removed using a dental drill, the dentin surface exhibited visible mechanical damage. Damage to the tooth surface was caused by the contact of the drill bit with the dentin surface. The SEM image shows dentinal tubules filled with the smear layer formed from elements of the mechanically prepared tooth. The visible cracks on the tooth surface were due to the intensive drying of the samples prior to the SEM analysis and not by mechanical actions (Figure 1A). The use of a dental curette for additional cleaning of the dentin surface after removal of the composite filling using a drill led to deeper cracks and grooves on the dentin surface. The curettage procedure did not remove the smear layer (Figure 1B).

### 3.2. EDTA Application on the Dentin Surface

**Figure 2:**
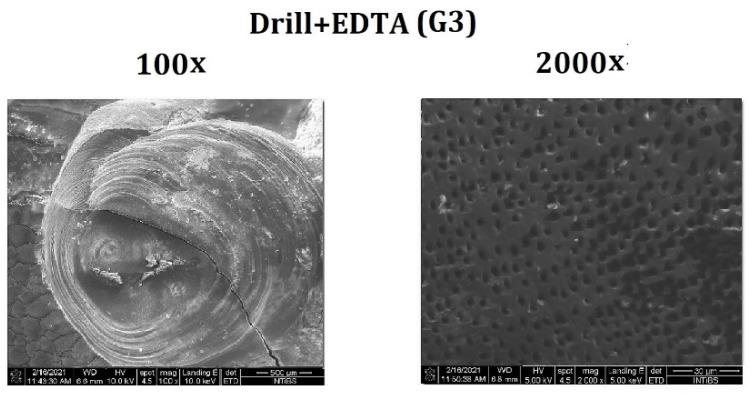
Dentin surface after the composite filling was removed using a diamond drill and 24% EDTA conditioning.

The use of 24% EDTA after the removal of the composite material using a diamond drill exposed dentinal tubules that were clearly visible at 2000× magnification. Inside the dentinal tubules, secondary residues of the activity of EDTA were present, mainly in the form of fine crystal structures formed by the drying of the samples before the SEM analysis. The lateral walls of the inside of the dentinal tubules showed loose collagen fibers forming the stroma of the dentin, which were released after chelation by EDTA. These changes were clearly visible at 2000× magnification (Figure 2).

### 3.3. Er:YAG Laser Applicaion on the Dentin Surface

**Figure 3:**
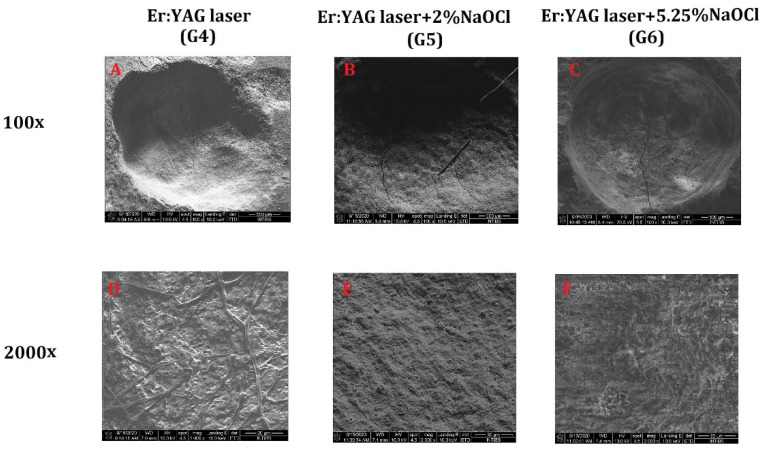
Dentin surface after the removal of the composite filling using the Er:YAG laser (**A**,**D**) and after additional dentin conditioning with 2% NaOCl (**B**,**E**) and 5.25% NaOCl (**C**,**F**).

The SEM analysis showed the regular shape of the cavity, which was formed by the removal of the composite filling using the Er:YAG laser. Partially denatured collagen fiber bundles at the bottom of the cavity were present. Moreover, the dentinal tubules under the layer of cross-linked collagen fibers were also exposed. No smear layer formation was observed (Figure 3A).

In the images of samples conditioned with 2% and 5.25% NaOCl, the structure image was similar to those of the samples irradiated with the Er:YAG laser alone. However, there was an evident secondary crystallization of NaOCl resulting from the procedures used for preparation of the material for observation in the SEM analysis. The sizes of NaOCl crystals were directly proportional to NaOCl concentration. However, dentin conditioning with NaOCl (Groups 5 and 6) did not increase the effect of dentinal tubule exposure compared to the Er:YAG laser alone (Group 4) (Figure 3B,C).

### 3.4. Semi-Quantitative Evaluation

The results of the semi-quantitative evaluation indicated superiority of preparation using an Er:YAG laser compared to the traditional approach: a drill or drill + curette. The use of drills generated an excessive amount of smear layer that was almost impossible to completely remove even by 24% EDTA conditioning. The use of the Er:YAG laser did not form any smear layer. The surfaces prepared with the Er:YAG laser had regular structures and open dentinal tubules. The images of the dentin surfaces after the use of the erbium laser were similar for all tooth samples under analysis. The results of the quantitative analysis indicated significant differences between the samples irradiated with the Er:YAG laser (G4, G5, G6) and those treated with the drill alone and in combination with the curette (G1, G2) *p* < 0.05 (Table 1).

## 4. Discussion

Open dentinal tubules (absence of smear layer) on the tooth surface are among the critical elements that determine the quality of fibroblast adhesion to the dental root surface [26]. To ensure fibroblast proliferation on the radicular dentin, its surface should have open dentinal tubules without the presence of the smear layer [10,27,28]. The smear layer is a layer composed of the collagen molecules and mineralized matrix that are present after mechanical tooth preparation [1]. Studies suggest that the smear layer may function as a barrier to the formation of the connective tissue attachment to the root surface [29]. During cavity preparation using a drill, the smear layer is present on the entire dentin surface, which was also confirmed by this study.

The results of this study indicate that mechanical preparation of dentin is associated with the smear layer formation. Furthermore, tooth surfaces prepared with the Er:YAG laser had a homogeneous structure without damage, along with open dentinal tubules (without smear layer) with visible denaturation of collagen fibers. However, the use of different sodium hypochlorite (NaOCl) solutions (2% and 5.25%) often used in dentistry did not increase the visibility of dentinal tubules. Smear layer formation is associated with contact preparation of the dentin, and it leads to the plugging of dentinal tubules [30,31]. The layer (thickness of 2–15 μm) is composed of organic and inorganic material, with particle sizes ranging from less than 1 μm to more than 15 μm; therefore, it can close (plug) dentinal tubules of different diameters. The smear layer is tightly bonded to the tooth surface, and it can be practically removed only by demineralizing solutions, such as EDTA, which was used in this study [32].

Various methods are used for removing the smear layer, e.g., chemical (37% orthophosphoric acid, EDTA, NaOCl, citric acid), ultrasonic and laser, or their combinations. Among the many chemicals that remove the smear layer, EDTA is widely used in dentistry. In their study, Demiryürek et al. [33] showed that the application of 17% EDTA followed by 5% NaOCl facilitates complete removal of the smear layer (opening of the dentinal tubules) by generating surface erosion. Moreover, other studies have confirmed the combination of EDTA with NaOCl as highly effective for smear layer removal [34,35]. Furthermore, Lo Giudice G et al. [36] proposed using protocols that applied ultrasound-activated EDTA alone or associated with orthophosphoric acid as the most effective in smear layer removal and cleansing of the dentinal surface. EDTA is a biocompatible compound that is well tolerated by tooth tissues, and it exhibits strong chelating properties. Unlike NaOCl, EDTA does not exhibit antimicrobial or dissolving activity in relation to organic tissues [37]. When the smear layer is removed, chelating agents demineralize the dentin, exposing collagen fibers. The depth of demineralization (approx. 1–6 µm) depends on the concentration and activity of the chelating agent [37,38]. The 24% EDTA applied for 2 min used in this study managed to sufficiently remove the smear layer formed. It should be noted, however, that EDTA does not affect decontamination of the dentin surface but leaves bacterial colonies on it, which may affect the formation of connective tissue attachment [11].

In their published study, Hibst and Keller presented SEM observations of the hard-tissue surface of the tooth after application of Er:YAG laser radiation [18,39]. These observations showed no damage to the hard tissues of the tooth. In other studies by Hibst and Keller [39], and also by Esteves-Oliveira M et al. [26], the following advantages of the preparation of hard tissues using the laser method were emphasized: a rough surface with no signs of demineralization, open dentinal tubules, the absence of the smear layer and cleanliness of the obtained surface. These findings are similar to the observations of this study. Kuhn et al. [40], meanwhile, indicated that during preparation of the dentin using an Er:YAG laser, there was denaturation of overlapping collagen fibers, which is also visible on SEM in this study. It should be noted that due to the higher percentage of water in the intertubular dentin than that in the peritubular dentin, erbium lasers ablate the intertubular dentin to a greater extent, which leads to formations in the microscopic surface of the dentin resembling protrusions (outgrowths). Therefore, optimal laser parameters set during composite filling removal and dentin conditioning are essential [41]. In the study, after composite filling removal with an Er:YAG laser, the pulse energy was decreased from 110 mJ to 80 mJ. Decreasing the pulse energy aimed to obtain a less rough and more homogeneous surface in the cavity and to reduce the denaturation effect in the collagen fibers. In addition, it should be underlined that after laser irradiation, we faced some water dehydration that impaired dentin strength and caused shrinkage of the collagen fibers. Thus, when preparing the tooth clinically in vivo, the surface of the dentin should be rehydrated by irrigating the tooth with the distilled water [1].

The use of an erbium laser, as indicated by the SEM images of the dentin, causes partial denaturation of the collagen fibers located in dentinal tubules [40]. The removal of denatured collagen fibers can be effected by the use of NaOCl [42]. Sodium hypochlorite (NaOCl) additionally shows strong bactericidal and antiviral activity. Due to its corrosive properties, NaOCl exhibits cytotoxicity to the oral mucosa and facial skin [43]. It lacks demineralizing properties, and, thus, it affects only the organic part of the smear layer [42]. According to studies by other authors, it is not possible to remove the smear layer with the use of NaOCl alone without chelators [44]. Nevertheless, NaOCl is a good complement to EDTA and citric acid because, in addition to dissolving the organic part of the smear layer, it leads to decontamination of the tooth surface [45]. In the present study we did not include the combination of NaOCl and EDTA, because the dentin surface irradiated by erbium laser was free of the smear layer. Only 2% and 5.25% NaOCl were used to check its ability to remove denatured fibers and open wider dentinal tubules. Some studies [40,46] used properties of NAOCl at different concentrations to clean and open dentinal tubules on the surface prepared with an Er:YAG laser. In this study, the use of NaOCl at concentrations of 2% and 5.25% as an additional step after the dentin surface preparation using the erbium laser did not change its microstructure (opening of dentinal tubules). Based on these results, cleaning the dentin surface with NaOCl to remove denatured collagen fibers and to better expose dental tubules is not recommended as an additional step in root preparation before a surgical recession coverage procedure.

An important limitation of in vitro study when using SEM is the difficulty in the discrimination of smear plugs and laminae limitantes in dentin after the demineralization and deproteinization of tooth samples [47]. Therefore, further studies should be conducted to evaluate the differentiation of the tag-like structures from laminae limitantes in demineralized and deproteinized specimens. Furthermore, given the relatively small number of test samples and disadvantages of an in vitro testing, further studies should examine the effectiveness of gingival recession coverage after root surface preparation using an Er:YAG laser in comparison with the use of a drill with additional chemical conditioning (e.g., EDTA + NaOCl combination) of dentin in vivo.

## 5. Conclusions

The use of an Er:YAG laser for composite filling removal (energy density: 14.01 J/cm^2^) with additional laser dentin conditioning (energy density: 10.19 J/cm^2^) results in a dentin surface with open dentin tubules, no smear layer and no mechanical damage. The main clinical relevance based on this in vitro experiment is that when preparing the dentin surface using an Er:YAG laser before recession coverage, additional NaOCl conditioning to achieve better exposure of dentinal tubule openings appears unnecessary.

## Figures and Tables

**Table 1:** The results of semi-quantitative studies concerning samples. Similar small (a, b, c, d) letters in a column indicate statistical significance between groups. (*p* < 0.05).

Group	Drill G1	Drill + Curette (G2)	Drill + EDTA (G3)	Er:YAG Laser (G4)	Er:YAG Laser + 2% NaOCl (G5)	Er:YAG Laser + 5.25% NaOCl (G6)
Quality of preparation (0–2)	1	1	1	2	2	2
Exposure of dentinal tubules (0–1)	0	0	1	1	1	1
Absence of smear layer (0–1)	0	0	1	1	1	1
Repeatability of results obtained (Similar sample image) (0–2)	0	0	1	2	2	2
Scoring	1 ^a,b,c^	1 ^a,b,c^	4 ^d^	6 ^a^	6 ^b^	6 ^c^
*p* Value	G4 vs. G2, G1 *p* < 0.05 G5 vs. G2, G1 *p* < 0.05 G6 vs. G2, G1 *p* < 0.05					

## Data Availability

The data presented in this study are available on request from the corresponding author.
